# The roles of interleukins in perfusion recovery after peripheral arterial disease

**DOI:** 10.1042/BSR20171455

**Published:** 2018-02-13

**Authors:** Lingdan Chen, Hanwei Liu, Mingjie Yuan, Wenju Lu, Jian Wang, Tao Wang

**Affiliations:** 1 State Key Laboratory of Respiratory Diseases, Guangzhou Institute of Respiratory Disease, The First Affiliated Hospital, Guangzhou Medical University, Guangzhou, Guangdong 510182, China; 2 Department of Cardiology, Renmin Hospital of Wuhan University, Wuhan 430071, China

**Keywords:** Angiogenesis, Arteriogenesis, Interleukin, Peripheral Arterial Disease

## Abstract

In peripheral arterial disease (PAD) patients, occlusions in the major arteries that supply the leg makes blood flow dependent on the capacity of neovascularization. There is no current medication that is able to increase neovascularization to the ischemic limb and directly treat the primary problem of PAD. An increasing body of evidence supports the notion that inflammation plays an important role in the vascular remodeling and perfusion recovery after PAD. Interleukins (ILs), a group of proteins produced during inflammation, have been considered to be important for angiogenesis and arteriogenesis after tissue ischemia. This review summarizes the latest clinical and experimental developments of the role of ILs in blood perfusion recovery after PAD.

## Introduction of peripheral arterial disease

Peripheral arterial disease (PAD) is caused by atherosclerosis that leads to occlusions of the arteries to the lower extremities. This affects more than 200 million people worldwide and puts them at risk for lower extremity amputation and death [1–3]. Over the past 20 years, the prevalence of PAD has continued, due to an increase in diabetes, smoking, and an aging patient population [4,6,7]. The primary cause of morbidity and mortality from PAD is due to the reduced blood flow to the lower extremities. Since total occlusions along the path of the sole major inflow artery to the leg(s) is common in symptomatic patients, the quantity of blood that can be delivered to the distal tissue becomes dependent on the extent of neovascularization, which is important to rebuild the vascular network in the ischemic extremity [5,8,9]. However, the mechanisms responsible for neovascularization after ischemia are not fully understood. In PAD patients, limb ischemia causes tissue hypoxia, which leads to the generation of hypoxia-inducible growth factors and the recruitment of inflammatory cells. These may work together to promote ischemia-induced neovascularization and vascular remodeling, which can be divided into two aspects [10–13]. First, new capillaries grow from pre-existing vessels and then form capillary networks to expand blood flow distribution in ischemic tissues downstream of the arterial occlusion, which is termed as angiogenesis [14]. Afterward, functional collateral arteries grow from pre-existing arterio-arteriolar anastomoses around the occlusion to allow greater in-flow to the distal ischemic tissue, which is termed as arteriogenesis [15]. Therefore, strategies to promote sustainable and functional blood flow after arterial occlusion in PAD should include the induction of both capillary angiogenesis and arteriogenesis [16,17]. Currently, peripheral vascular intervention is preferred as a first-line treatment for revascularization for severe PAD patients, but many patients have no revascularization options, because of limited access to catheterization labs, especially in developing countries. While some pharmaceutical therapies with statins, and antiplatelet agents have shown some efficacy in preventing artery occlusion in PAD patients; however, no pharmacological agents have been able to increase neovascularization to the ischemic limb resulting from the arterial occlusions after PAD [18,19].

## Inflammation, interleukins, and neovascularization

In PAD, hypoxia in ischemic limbs typically initiates inflammation after tissue damage. Both experimental models and patients with PAD suggest that inflammation is important for angiogenesis and perfusion recovery after limb ischemia. Depletion of T cells, specifically the subtypes of CD4+, CD8+, regulatory T cells (T_regs_) or Th17 cells have been reported to impair angiogenesis and perfusion recovery in experimental PAD models [20–23]. Natural killer (NK) cells also appear to play a role in the hind limb ischemia model as indicated by impaired collateral artery growth after NK cell depletion in a mouse PAD model [24]. Using a macrophage-specific, near-IR fluorophore; Yoo et al. [25] – found an increased number of macrophages in the ischemic hind limb, compared with the non-ischemic side in a mice PAD model. Since macrophages are best known for their clear link to arteriogenesis [26–28], they could play a role in angiogenesis [29], particularly when they are of the M2 phenotype [27,30].

Interleukins (IL) are a group of signaling proteins produced and secreted during inflammation, and participate in communication amongst leukocytes regulating numerous biological processes and immune responses. Recent evidence from animal models and studies in patients with PAD suggest that a number of ILs or IL receptors are increased in muscle tissue after limb ischemia. Some of the ILs are increased in the circulation indicating that PAD initiates a systemic response of inflammation after limb ischemia [31]. Interaction of ILs and their receptors in a variety of cells, including endothelial cells, T cells, and macrophages modulate angiogenesis and arteriogenesis in the ischemic lower extremity [32–35]. Some of the ILs have shown promising effects in perfusion recovery improvement in preclinical PAD models. Herein, we review experimental results and clinical data of the most important ILs in vascular remodeling and perfusion recovery after PAD.

## Specific ILs

### IL-10

IL-10 is primarily produced by macrophages/monocytes and, to a lesser extent, T and B lymphocytes, and signals through binding to a specific receptor complex to induce pleiotropic effects in inflammation and immune regulation [36,37]. IL-10 is generally considered as an anti-inflammatory cytokine. In a mouse PAD model, IL-10 is significantly up-regulated in the ischemic limbs [38]. Silvestre et al. [38] reported that IL-10 depletion resulted in increased angiogenesis and better perfusion recovery; while IL-10 overexpression using plasmid transfection impaired perfusion recovery and reduced angiogenesis. However, a more recent study showed that depletion of T_regs_ by using a CD25 antibody resulted in a lesser extent of angiogenesis, arteriogenesis, and impaired perfusion recovery in a mouse PAD model, which was associated with reduced IL-10 levels. Adoptive T_reg_ transfer increased perfusion recovery and angiogenesis, and these effects were abolished when IL-10 was neutralized by an IL-10 antibody [23]. These two studies indicate that the effect of IL-10 in perfusion recovery is bidirectional under different circumstances. It is not surprising because ILs are versatile molecules that induce different effects under different circumstances. The latter study being performed in the context of T_reg_ transplantation, whereas the earlier study being performed in mice without T_reg_ modulation. Clinical data showed that circulating IL-10 was slightly higher in PAD patients when compared with a healthy control group, although not statistically significant [39].

### IL-11

IL-11 is a multifunctional cytokine with pleiotropic effects on multiple tissues, including the promotion of megakaryocyte maturation, thrombopoiesis, and protection of endothelial cell viability against injuries and death [40]. As a signaling molecule, IL-11 functions through its receptor, termed as IL-11 receptor α (IL-11Rα). Interestingly, IL-11Rα is highly expressed in the CD34^+^/vascular endothelial growth factor (VEGF) receptor (VEGFR) 2^+^ mononuclear cells, which are a type of progenitor cells that are important for angiogenesis and arteriogenesis [41]. A recent study on mouse hind limb ischemia demonstrated that recombinant human IL-11 increased the number of CD34+/VEGFR2+ mononuclear cells in the blood and also the perivascular region of ischemic hind limbs. In the ischemic limb, CD34+/VEGFR2+ cells differentiated to endothelial cells and are important components of new blood vessels that provided blood supply to the ischemic tissue. In addition, cytokines and growth factors secreted from CD34+/VEGFR2+ cells activated signal transducer and activator of a transcription 3 (STAT3)-dependent anti-apoptotic pathway, which is important to sustain limb function and reduce tissue necrosis [41]. Because recombinant IL-11 has been used for other clinical conditions [42], these data may suggest that IL-11 could potentially be used as an adjunctive treatment for PAD.

### IL-17

IL-17 is a pro-inflammatory cytokine produced by a group of CD4^+^ T-helper cells termed as Th17 cells, and acts as a potent mediator in delayed-type reactions by increasing chemokine production and recruiting leukocytes to the site of inflammation. Although Th17 cells have been reported to play an important role in the pathophysiology of various diseases including atherosclerosis and hypertension [33], a recent study showed that Th17 cells are important for angiogenesis after PAD. Th17 cell depletion or IL-17 blockage resulted in impaired angiogenesis as well as reduced VEGF-A production. In addition, the specific cytokine of Th17 cells and IL-17 expression is up-regulated after hind limb ischemia [21]. Interestingly, clinical data showed that the serum IL-17 levels were associated with the severity of atherosclerotic plaque lesions which initiates the development of PAD [33]. Collectively, this may suggest that although IL-17 contributes to the development of PAD, it is adaptively up-regulated in the ischemic tissue, and also contributes to the perfusion recovery and angiogenesis after limb ischemia.

### IL-18

IL-18, also known as interferon-γ inducing factor, is a pro-inflammatory cytokine. It works by binding to the IL-18 receptor and stimulates interferon-γ and other cytokines production, and thus, enhances immune responses [43]. Interestingly, an endogenous protein, termed as IL-18 binding protein (IL-18BP), prevents the binding of IL-18 to its receptor, and thus inhibits IL-18 interaction with its receptor [44]. IL-18BP is expressed and secreted by mononuclear cells and inhibits IL-18 signaling. In PAD models, Mallat et al. reported endogenous IL-18 is an inhibitor of ischemia-induced neovascularization in the mouse hind limb [45] When treated *in vivo* with IL-18BP, enhanced neovascularization in the ischemic hind limb was seen by promoting VEGF production and by activating the protein kinase B (Akt) pathway [45]. Clinical data showed that serum IL-18 levels were a predictor of cardiovascular mortality. In PAD patients with type 2 diabetes, the IL-18 level is significantly higher than in non-diabetic PAD patients [46], which may suggest that IL-18 contributes to the extent of impaired perfusion recovery induced in diabetic patients with PAD.

### IL-19 and IL-20

IL-19 is an IL-10 family member and is generally considered as an anti-inflammatory cytokine. A recent report shows that IL-19 and its receptor, the IL-20 receptor, were expressed in endothelial cells, and IL-19 expression was up-regulated when endothelial cells were stimulated by basic fibroblast growth factor (b-FGF) [47]. *In vitro* experiments indicate that IL-19 promoted endothelial cell tube formation and angiogenesis. In experimental PAD, depletion of IL-19 resulted in impaired perfusion recovery, while exogenous IL-19 treatment increased capillary density and perfusion recovery [48]. The mechanisms of IL-19 on perfusion recovery included inducing macrophage M2 polarization, direct angiogenic effects on endothelial cells and increasing VEGF-A production [48,49]. In LDL receptor knockout mice, IL-19 decreased atherosclerosis and increases angiogenesis. This is the first IL which has been reported to be both pro-angiogenic and anti-atherosclerotic [50]. Interestingly, there are two ligands that activate the IL-20 receptor, in addition to IL-19, the other one is IL-20. In a rat PAD model, IL-20 increased the collateral artery network and improved perfusion recovery and muscle function, which is similar to IL-19 [51].

### IL-21

The IL-21 receptor (IL-21R) belongs to the type I cytokine receptor family which forms a heterodimeric receptor complex with the common γ chain [52]. Because of its immune regulating effects, IL-21 delivery has become an area of active research. Indeed, the administration of recombinant IL-21 is currently being explored in a host of human diseases in at least 12 clinical trials [52]. In both mice and human ischemic hind limbs, IL-21R was up-regulated in endothelial cells, and activation of endothelial IL-21R by its sole ligand (IL-21) has been shown to promote angiogenesis via STAT3 activation. However, the effects of IL21 on angiogenesis are complicated: in a tumor environment with abundant growth IL-21R activation was shown to be angiostatic, and IL-21 administration decreased tumor vascular density and tumor size in mice bearing EG7 tumor cells via STAT1 activation [53]. Taken together, these studies suggest that IL-21 induces either angiostasis or angiogenesis under different conditions, by activating different pathways.

## Conclusion and perspectives

**Figure 1:**
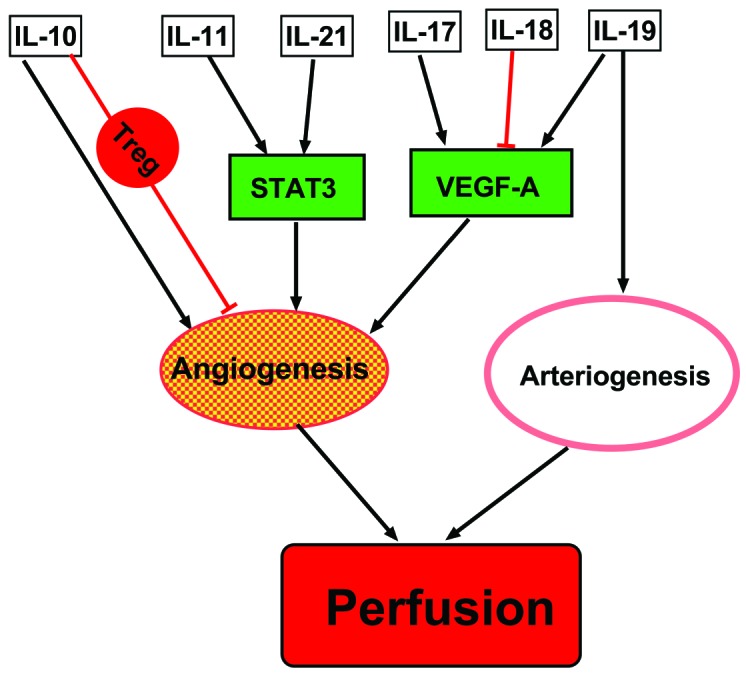
Molecular and cellular mechanisms of ILs actions on angiogenesis in ischemic limb after PAD VEGF and STAT3 are the two main pathways involved in the process of angiogenesis modulated by ILs. Macrophages is polarized by IL-19, which leads to increased arteriogenesis. Both angiogenesis and arteriogenesis contribute to perfusion recovery after PAD.

Accumulating evidence reveals that ILs play a crucial role in perfusion recovery after PAD through their influence on angiogenesis and arteriogenesis via regulating STAT3 and VEGF pathways (Figure 1). Notably, the IL-based therapeutic approach has had a remarkable outcome in a variety of experimental PAD models. There are some human studies that indicate ILs and IL receptors are highly altered in the plasma or tissue of PAD patients in a similar manner as found in experimental models, which suggests the possible initiation of clinical studies of human PAD. Given the complex network amongst the cells, ILs and their signaling pathways (Figure 1), targetting one specific factor might not prove successful in a clinical setting. Since some of the ILs have been used in other clinical conditions, developing a cocktail of multiple factors might provide a novel therapy for PAD.

## Abbreviations

IL: interleukin
IL-18BP: IL-18 binding protein
IL-21R: IL-21 receptor
IL-11Rα: IL-11 receptor α
NK: natural killer
PAD: peripheral arterial disease
STAT3: signal transducer and activator of a transcription 3
T_reg_: regulatory T cell
VEGF: vascular endothelial growth factor
VEGFR2+: VEGF receptor 2+

## References

[1] Guerchet M., Aboyans V., Mbelesso P., Mouanga A.M., Salazar J., Bandzouzi B. (2012) Epidemiology of peripheral artery disease in elder general population of two cities of Central Africa: Bangui and Brazzaville. Eur. J. Vasc. Endovasc. Surg. 44, 164–169 10.1016/j.ejvs.2012.05.019 22705162

[2] Criqui M.H. and Aboyans V. (2015) Epidemiology of peripheral artery disease. Circ. Res. 116, 1509–1526 10.1161/CIRCRESAHA.116.303849 25908725

[3] Fowkes F.G., Aboyans V., Fowkes F.J., McDermott M.M., Sampson U.K. and Criqui M.H. (2017) Peripheral artery disease: epidemiology and global perspectives. Nat. Rev. Cardiol. 14, 156–170 10.1038/nrcardio.2016.179 27853158

[4] Emdin C.A., Anderson S.G., Callender T., Conrad N., Salimi-Khorshidi G., Mohseni H. (2015) Usual blood pressure, peripheral arterial disease, and vascular risk: cohort study of 4.2 million adults. BMJ 351, h4865 10.1136/bmj.h4865 26419648 PMC4586462

[5] Annex B.H. and Beller G.A. (2016) Towards the development of novel therapeutics for peripheral artery disease. Trans. Am. Clin. Climatol. Assoc. 127, 224–234 28066055 PMC5216482

[6] Fowkes F.G., Rudan D., Rudan I., Aboyans V., Denenberg J.O., McDermott M.M. (2013) Comparison of global estimates of prevalence and risk factors for peripheral artery disease in 2000 and 2010: a systematic review and analysis. Lancet 382, 1329–1340 10.1016/S0140-6736(13)61249-0 23915883

[7] Thiruvoipati T., Kielhorn C.E. and Armstrong E.J. (2015) Peripheral artery disease in patients with diabetes: Epidemiology, mechanisms, and outcomes. World J. Diabetes 6, 961–969 10.4239/wjd.v6.i7.961 26185603 PMC4499529

[8] Ko S.H. and Bandyk D.F. (2014) Therapeutic angiogenesis for critical limb ischemia. Semin. Vasc. Surg. 27, 23–31 10.1053/j.semvascsurg.2014.10.001 25812756

[9] Annex B.H. (2013) Therapeutic angiogenesis for critical limb ischaemia. Nat. Rev. Cardiol. 10, 387–396 10.1038/nrcardio.2013.70 23670612

[10] Troidl K. and Schaper W. (2012) Arteriogenesis versus angiogenesis in peripheral artery disease. Diabetes Metab. Res. Rev. 28, 27–29 10.1002/dmrr.2232 22271719

[11] Terjung R.L. and Yang H.T. (2007) Exercise-induced angiogenesis and arteriogenesis. FASEB J. 21, A79–A79

[12] Carmeliet P. (2000) Mechanisms of angiogenesis and arteriogenesis. Nat. Med. 6, 389–395 10.1038/74651 10742145

[13] Buschmann I. and Schaper W. (1999) Arteriogenesis versus angiogenesis: Two mechanisms of vessel growth. News Physiol. Sci. 14, 121–125 11390835 10.1152/physiologyonline.1999.14.3.121

[14] Semenza G.L. (2007) Vasculogenesis, angiogenesis, and arteriogenesis: Mechanisms of blood vessel formation and remodeling. J. Cell. Biochem. 102, 840–847 10.1002/jcb.21523 17891779

[15] Grundmann S., Piek J.J., Pasterkamp G. and Hoefer I.E. (2007) Arteriogenesis: basic mechanisms and therapeutic stimulation. Eur. J. Clin. Invest. 37, 755–766 10.1111/j.1365-2362.2007.01861.x 17764463

[16] Heil M., Eitenmuller I., Schmitz-Rixen T. and Schaper W. (2006) Arteriogenesis versus angiogenesis: similarities and differences. J. Cell. Mol. Med. 10, 45–55 10.1111/j.1582-4934.2006.tb00290.x 16563221 PMC3933101

[17] Meisner J.K., Song J., Annex B.H. and Price R.J. (2013) Myoglobin overexpression inhibits reperfusion in the ischemic mouse hindlimb through impaired angiogenesis but not arteriogenesis. Am. J. Pathol. 183, 1710–1718 10.1016/j.ajpath.2013.08.005 24095922 PMC5746951

[18] Annex B.H. (2013) Therapeutic angiogenesis for critical limb ischaemia. Nat. Rev. Cardiol. 10, 387–396 10.1038/nrcardio.2013.70 23670612

[19] Hirsch A.T. (2006) Treatment of peripheral arterial disease - extending “intervention” to “therapeutic choice”. New Engl. J. Med. 354, 1944–1947 10.1056/NEJMe068037 16672707

[20] Stabile E., Burnett M.S., Watkins C., Kinnaird T., Bachis A., la Sala A. (2003) Impaired arteriogenic response to acute hindlimb ischemia in CD4-knockout mice. Circulation 108, 205–210 10.1161/01.CIR.0000079225.50817.71 12821542

[21] Hata T., Takahashi M., Hida S., Kawaguchi M., Kashima Y., Usui F. (2011) Critical role of Th17 cells in inflammation and neovascularization after ischaemia. Cardiovasc. Res. 90, 364–372 10.1093/cvr/cvq397 21156823

[22] Pellegrin M., Bouzourene K., Poitry-Yamate C., Mlynarik V., Feihl F., Aubert J.F. (2014) Experimental peripheral arterial disease: new insights into muscle glucose uptake, macrophage, and T-cell polarization during early and late stages. Physiol. Rep. 2, e00234 10.1002/phy2.234 24744903 PMC3966252

[23] Sharir R., Semo J., Shaish A., Landa-Rouben N., Entin-Meer M., Keren G. (2014) Regulatory T cells influence blood flow recovery in experimental hindlimb ischaemia in an IL-10-dependent manner. Cardiovasc. Res. 103, 585–596 10.1093/cvr/cvu159 24966183

[24] van Weel V., Toes R.E., Seghers L., Deckers M.M., de Vries M.R., Eilers P.H. (2007) Natural killer cells and CD4+ T-cells modulate collateral artery development. Arterioscler. Thromb. Vasc. Biol. 27, 2310–2318 10.1161/ATVBAHA.107.151407 17717295

[25] Yoo J.S., Das R.K., Jow Z.Y. and Chang Y.T. (2014) *In vivo* detection of macrophage recruitment in hind-limb ischemia using a targeted near-infrared fluorophore. PLoS ONE 9, e103721 10.1371/journal.pone.0103721 25072508 PMC4114964

[26] Mantovani A., Biswas S.K., Galdiero M.R., Sica A. and Locati M. (2013) Macrophage plasticity and polarization in tissue repair and remodelling. J. Pathol. 229, 176–185 10.1002/path.4133 23096265

[27] Takeda Y., Costa S., Delamarre E., Roncal C., Leite de Oliveira R., Squadrito M.L. (2011) Macrophage skewing by Phd2 haplodeficiency prevents ischaemia by inducing arteriogenesis. Nature 479, 122–126 10.1038/nature10507 21983962 PMC4659699

[28] Yang B.L., Wu S., Wu X., Li M.B., Zhu W., Guan Y. (2013) Effect of shunting of collateral flow into the venous system on arteriogenesis and angiogenesis in rabbit hind limb. Acta Histochem. Cytochem. 46, 1–10 10.1267/ahc.12025 23554534 PMC3596601

[29] Rajagopalan S., Mohler E.R. III, Lederman R.J., Mendelsohn F.O., Saucedo J.F., Goldman C.K. (2003) Regional angiogenesis with vascular endothelial growth factor in peripheral arterial disease: a phase II randomized, double-blind, controlled study of adenoviral delivery of vascular endothelial growth factor 121 in patients with disabling intermittent claudication. Circulation 108, 1933–1938 10.1161/01.CIR.0000093398.16124.29 14504183

[30] Jetten N., Donners M.M., Wagenaar A., Cleutjens J.P., van Rooijen N., de Winther M.P. (2013) Local delivery of polarized macrophages improves reperfusion recovery in a mouse hind limb ischemia model. PLoS ONE 8, e68811 10.1371/journal.pone.0068811 23894348 PMC3722193

[31] Wang T., Cunningham A., Houston K., Sharma A.M., Chen L., Dokun A.O. (2016) Endothelial interleukin-21 receptor up-regulation in peripheral artery disease. Vasc. Med. 21, 99–104 10.1177/1358863X15621798 26705256 PMC5347532

[32] David A., Saitta S., De Caridi G., David T., Noto A., Minciullo P.L. (2014) Different serum levels of interleukin-23 in patients affected by peripheral arterial disease. Vascular 22, 471–472 10.1177/1708538113498590 24067793

[33] Karbach S., Croxford A.L., Oelze M., Schuler R., Minwegen D., Wegner J. (2014) Interleukin 17 drives vascular inflammation, endothelial dysfunction, and arterial hypertension in psoriasis-like skin disease. Arterioscler. Thromb. Vasc. Biol. 34, 2658–2668 10.1161/ATVBAHA.114.304108 25341795

[34] Wang T., Cunningham A., Dokun A.O., Hazarika S., Houston K., Chen L. (2015) Loss of interleukin-21 receptor activation in hypoxic endothelial cells impairs perfusion recovery after hindlimb ischemia. Arterioscler. Thromb. Vasc. Biol. 35, 1218–1225 10.1161/ATVBAHA.115.305476 25838422 PMC4865891

[35] Wang T., Cunningham A., Houston K., Sharma A.M., Chen L., Dokun A.O. (2016) Endothelial interleukin-21 receptor up-regulation in peripheral artery disease. Vasc. Med. 21, 99–104 10.1177/1358863X15621798 26705256 PMC5347532

[36] Moore K.W., de Waal Malefyt R., Coffman R.L. and O’Garra A. (2001) Interleukin-10 and the interleukin-10 receptor. Annu. Rev. Immunol. 19, 683–765 10.1146/annurev.immunol.19.1.683 11244051

[37] Peng H., Wang W., Zhou M., Li R., Pan H.F. and Ye D.Q. (2013) Role of interleukin-10 and interleukin-10 receptor in systemic lupus erythematosus. Clin. Rheumatol. 32, 1255–1266 10.1007/s10067-013-2294-3 23708831

[38] Silvestre J.S., Mallat Z., Duriez M., Tamarat R., Bureau M.F., Scherman D. (2000) Antiangiogenic effect of interleukin-10 in ischemia-induced angiogenesis in mice hindlimb. Circ Res. 87, 448–452 10.1161/01.RES.87.6.448 10988235

[39] Cauley J.A., Kassem A.M., Lane N.E., Thorson S. and (2016) Prevalent peripheral arterial disease and inflammatory burden. BMC Geriatr. 16, 213 10.1186/s12877-016-0389-9 27938334 PMC5148838

[40] Kirkiles-Smith N.C., Mahboubi K., Plescia J., McNiff J.M., Karras J., Schechner J.S. (2004) IL-11 protects human microvascular endothelium from alloinjury *in vivo* by induction of survivin expression. J. Immunol. 172, 1391–1396 10.4049/jimmunol.172.3.1391 14734714

[41] Aitsebaomo J., Srivastava S., Zhang H., Jha S., Wang Z., Winnik S. (2011) Recombinant human interleukin-11 treatment enhances collateral vessel growth after femoral artery ligation. Arterioscler. Thromb. Vasc. Biol. 31, 306–312 10.1161/ATVBAHA.110.216986 21071685 PMC3025756

[42] Gordon M.S., McCaskill-Stevens W.J., Battiato L.A., Loewy J., Loesch D., Breeden E. (1996) A phase I trial of recombinant human interleukin-11 (neumega rhIL-11 growth factor) in women with breast cancer receiving chemotherapy. Blood 87, 3615–3624 8611685

[43] Rabkin S.W. (2009) The role of interleukin 18 in the pathogenesis of hypertension-induced vascular disease. Nat. Clin. Pract. Cardiovasc. Med. 6, 192–199 10.1038/ncpcardio1453 19234499

[44] Dinarello C.A., Novick D., Kim S. and Kaplanski G. (2013) Interleukin-18 and IL-18 binding protein. Front. Immunol. 4, 289 10.3389/fimmu.2013.00289 24115947 PMC3792554

[45] Mallat Z., Silvestre J.S., Le Ricousse-Roussanne S., Lecomte-Raclet L., Corbaz A., Clergue M. (2002) Interleukin-18/interleukin-18 binding protein signaling modulates ischemia-induced neovascularization in mice hindlimb. Circ. Res. 91, 441–448 10.1161/01.RES.0000033592.11674.D8 12215494

[46] Deser S.B., Bayoglu B., Besirli K., Cengiz M., Arapi B., Junusbekov Y. (2016) Increased IL18 mRNA levels in peripheral artery disease and its association with triglyceride and LDL cholesterol levels: a pilot study. Heart Vessels 31, 976–984 10.1007/s00380-015-0753-2 26438531

[47] Jain S., Gabunia K., Kelemen S.E., Panetti T.S. and Autieri M.V. (2011) The anti-inflammatory cytokine interleukin 19 is expressed by and angiogenic for human endothelial cells. Arterioscler. Thromb. Vasc. Biol. 31, 167–175 10.1161/ATVBAHA.110.214916 20966397 PMC3005139

[48] Richards J., Gabunia K., Kelemen S.E., Kako F., Choi E.T. and Autieri M.V. (2015) Interleukin-19 increases angiogenesis in ischemic hind limbs by direct effects on both endothelial cells and macrophage polarization. J. Mol. Cell Cardiol. 79, 21–31 10.1016/j.yjmcc.2014.11.002 25450612 PMC4301995

[49] Gabunia K. and Autieri M.V. (2015) Interleukin-19 can enhance angiogenesis by Macrophage Polarization. Macrophage 2, e562 26029742 10.14800/macrophage.562 PMC4447484

[50] Ellison S., Gabunia K., Kelemen S.E., England R.N., Scalia R., Richards J.M. (2013) Attenuation of experimental atherosclerosis by interleukin-19. Arterioscler. Thromb. Vasc. Biol. 33, 2316–2324 10.1161/ATVBAHA.113.301521 23950143 PMC3950941

[51] Tritsaris K., Myren M., Ditlev S.B., Hubschmann M.V., van der Blom I., Hansen A.J. (2007) IL-20 is an arteriogenic cytokine that remodels collateral networks and improves functions of ischemic hind limbs. Proc. Natl. Acad. Sci. U.S.A. 104, 15364–15369 10.1073/pnas.0707302104 17878297 PMC1978488

[52] Spolski R. and Leonard W.J. (2014) Interleukin-21: a double-edged sword with therapeutic potential. Nat. Rev. Drug Discov. 13, 381–393 10.1038/nrd4296 24751819

[53] Castermans K., Tabruyn S.P., Zeng R., van Beijnum J.R., Eppolito C., Leonard W.J. (2008) Angiostatic activity of the antitumor cytokine interleukin-21. Blood 112, 4940–4947 10.1182/blood-2007-09-113878 18515660 PMC6561391

